# Recommendations of the Polish Zebrafish Society on the use of the zebrafish (*Danio rerio*) model in biomedical research

**DOI:** 10.3389/abp.2026.16545

**Published:** 2026-05-22

**Authors:** Piotr Podlasz, Marta Migocka-Patrzałek, Tomasz K. Prajsnar, Anna Sarosiak, Przemko Tylzanowski

**Affiliations:** 1 Department of Pathophysiology, Forensic Veterinary Medicine and Administration, Faculty of Veterinary Medicine, University of Warmia and Mazury in Olsztyn, Olsztyn, Poland; 2 Department of Animal Developmental Biology, Faculty of Biological Sciences, University of Wrocław, Wrocław, Poland; 3 Department of Evolutionary Immunology, Institute of Zoology and Biomedical Research, Faculty of Biology, Jagiellonian University, Kraków, Poland; 4 University Center for Women’s and Newborn Health, Medical University of Warsaw, Warszawa, Poland; 5 Skeletal Biology and Engineering Research Center, Department of Development and Regeneration, KU Leuven, Leuven, Belgium; 6 Laboratory of Molecular Genetics, Department of Biomedical Sciences, Medical University of Lublin, Lublin, Poland

**Keywords:** 3Rs principles, biomedical research, zebrafish, Danio rerio, Polish Zebrafish Society, toxicology, oncology, translational research

## Abstract

The zebrafish (*Danio rerio*) has become a widely adopted vertebrate model in biomedical research, offering high translational value while supporting the principles of Replacement, Reduction, and Refinement (3Rs). Here, the Polish Zebrafish Society presents comprehensive, field-specific recommendations for the responsible and effective use of zebrafish across major areas of biomedical research. The document summarizes the scientific rationale and experimental advantages of zebrafish-based approaches in oncology, toxicology, neurology and neuropsychiatry, metabolic diseases, immunology, cardiology, and genetic disease modelling. The model’s key strengths include rapid development, optical transparency, genetic tractability, and strong conservation of molecular and physiological pathways relevant to human disease. These features enable real-time *in vivo* analysis of pathological processes, high-throughput pharmacological and toxicological screening, and functional validation of disease-associated genes. By integrating ethical considerations with robust experimental evidence, these recommendations aim to promote standardized implementation of zebrafish models in academic research, pharmaceutical development, and regulatory science. Broad adoption of zebrafish-based approaches can accelerate preclinical discovery, enhance translational relevance, and substantially reduce reliance on higher vertebrate models.

## Introduction

Responsibility for animal welfare is an essential part of scientific research involving the use of *in vivo* model organisms. This has led to the development of the 3Rs concept of Replacement, Reduction, and Refinement. The aim was to provide ethical guidelines for minimizing animal use and suffering in scientific research, while maintaining experimental validity. Recent developments have moved this concept further, proposing a fourth R (Responsibility) and a fifth R (Rehoming) (Harrell et al., 2024; Lauwereyns et al., 2024; MacArthur Clark, 2018).

Replacement strategies involve shifting experimental approaches away from higher vertebrates, such as mice and rats, towards animals that are evolutionarily less complex. One such approach relies on the zebrafish (*Danio rerio*), which has gained widespread popularity over the past two decades. While rodent models remain the most frequently used vertebrate systems, bibliometric trends suggest that the number of zebrafish-based studies is increasing rapidly, approaching and potentially exceeding the use of rat models in some research areas (Bedell et al., 2025; Choi et al., 2021).

Zebrafish is a small freshwater fish with approximately 70% of genetic homology with humans, and 80% of human disease-linked genes have orthologs in zebrafish (Howe et al., 2013). Zebrafish represent a conserved vertebrate body plan and share the same fundamental principles of organ development, tissue organization, and physiological regulation as mammals. While certain organs display species-specific anatomical differences, most zebrafish organs have clear human counterparts that arise through homologous developmental pathways. Significantly, many cellular processes relevant to human disease, including signal transduction, endocrine regulation, immune responses, and xenobiotic metabolism, are highly conserved. The zebrafish possess functional orthologs of key drug-metabolizing enzymes and transporters, enabling meaningful assessment of pharmacokinetics, efficacy, and toxicity at the whole-organism level. These features underpin the strong translational relevance of the zebrafish model despite its evolutionary distance from mammals. Zebrafish also have other advantages compared with higher vertebrates. Fertilization and embryonic development occur outside the maternal organism, embryos develop very rapidly with most organs forming within 24 h after fertilization, and they are transparent for the first few days, enabling real-time observation of organogenesis (Kimmel et al., 1995). Fish reach sexual maturity in approximately 3 months. They are small in size and easy to maintain in laboratory conditions, exhibit high fecundity with a single pair producing hundreds of eggs per week, and incur low maintenance costs compared with mammalian models. In addition, advanced molecular research techniques such as transgenesis, Cre-lox and CRISPR/Cas9 have been adopted for use with zebrafish enabling the modelling of human diseases and high-throughput *in vivo* screening experiments. Last but not least, their transparency has led to the development of very sophisticated whole-organism *in vivo* imaging techniques.

The Polish Zebrafish Society (pol. Polskie Towarzystwo “Zebrafish”) therefore recommends the use of zebrafish embryos and larvae up to the stage preceding independent feeding for *in vivo* experiments. This recommendation is consistent with EU Directive 2010/63/EU, which defines the scope of protection with reference to independently feeding larval forms; accordingly, developmental stages of fish prior to the onset of independent feeding are outside the Directive’s formal scope. In zebrafish, this stage is commonly reached at approximately 5 days post-fertilization (dpf), although the exact timing should be interpreted in relation to developmental and husbandry conditions. These principles have been fully incorporated into Polish national legislation through the Act of 15 January 2015 on the protection of animals used for scientific or educational purposes (Journal of Laws 2015, item 266, as amended), which likewise excludes embryonic and larval developmental stages of fish prior to independent feeding from the statutory scope of protection. Accordingly, the use of zebrafish embryos and early larvae may, under Polish and EU law, constitute an alternative to classical experiments performed on protected animal stages. This approach can contribute to the replacement or reduction of the use of commonly employed mammalian models, including mice and rats, in research where scientifically appropriate.

In scientifically justified situations in which no suitable alternative approach is available to obtain reliable results the Polish Zebrafish Society considers the use of later developmental stages or adult zebrafish to be justified, provided that such studies are conducted in accordance with applicable legal and ethical requirements. In such cases, the required approval from the competent Local Ethics Committee for Animal Experiments must be obtained in compliance with the applicable regulations.

The zebrafish has become a core animal model thanks to its unique combination of external and rapid development and transparent body in the early stages. It is widely used in developmental biology as it allows genetics, live imaging and quantitative multi-omics analysis to be integrated within a single vertebrate system. Zebrafish provide insight into the epigenetic regulation of lineage decisions, organogenesis, and the control of hormones and the endocrine system (Dawid, 2004; Kemmler et al., 2021; 2021; Lazcano et al., 2023; Marchione et al., 2021; Meyers, 2018; Vacaru et al., 2014). Alongside technologies that enable transcriptional analysis across cell populations, the characteristics of the zebrafish make it possible to create a comprehensive transcriptional atlas of early zebrafish development (Daniocell; Sur et al., 2023). Community efforts, such as large-scale mutagenesis screens and ZFIN repositories, provide open access to maps of vertebrate regulators. These efforts provide a solid foundation for new discoveries in the field (Baranasic et al., 2022).

**FIGURE 1:**
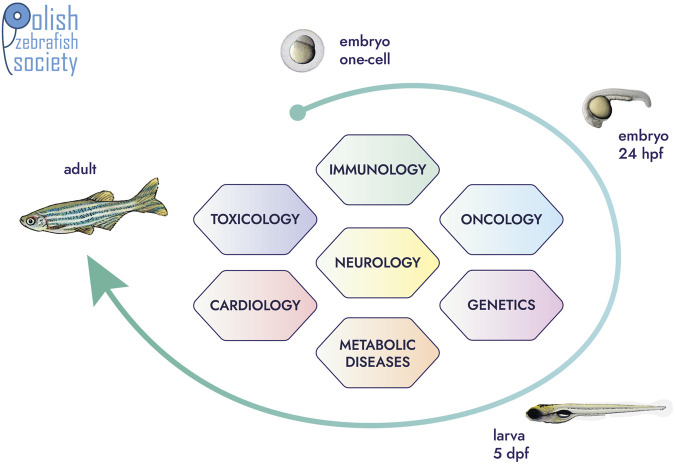
Zebrafish embryos, larvae and adults are used in multiple fields of biomedical research. The use of zebrafish as a model are common in oncology, toxicology, neurology, metabolic diseases, immunology, cardiology and genetic diseases. hpf–hours post fertilization, dpf - days post fertilization.

The following sections present field-specific recommendations and literature-based examples demonstrating the utility of zebrafish embryos and larvae across oncology, toxicology, and neurology (including neuropsychiatry), metabolic diseases, immunology, cardiology, and genetic diseases (Figure 1).

## Oncology

The Polish Zebrafish Society recommends using the human-zebrafish xenograft model for rapid screening in oncology research. This strategy involves implantation of human cancer cells, derived from either established cell lines or patients, into zebrafish embryos or larvae. Embryos and larvae do not have a fully functional immune system at this stage of development. The absence of an immune response in the early days of zebrafish larvae’s lives enables the engraftment and proliferation of human cells without the need for immunosuppression.

The transparent body of the larvae allows direct, *in vivo* visualization of tumor development and associated processes, such as angiogenesis, at single-cell resolution. Moreover, in contrast to mouse models, human cancer cells in zebrafish readily migrate and form metastases. This enables the assessment of the effects of drugs on this critical aspect of tumor biology under *in vivo* conditions (Fior et al., 2017; Pruvot et al., 2011). Xenograft experiments in zebrafish can be completed in less than 1 week, whereas analogous studies in mice typically require several weeks. This substantially reduces research costs and accelerates the acquisition of clinically relevant results. The model, commonly referred to as the “Avatar” approach, is already being applied in clinical contexts, for example, to optimize chemotherapy regimens as part of personalized medicine strategies (Costa et al., 2020; Lunina et al., 2024) although its clinical application remains under active validation. This approach involves short-term assays using zebrafish larvae that have been implanted with tumor cells derived from patients. It enables the *in vivo* assessment of tumor sensitivity to different chemotherapy regimens within a timeframe of several days. In a translational study involving patients with breast cancer, complete concordance was demonstrated between the responses observed in the larval model and the actual clinical responses to treatment. This confirms the high predictive value of the approach and its potential utility in personalized medicine (Mendes et al., 2025). Importantly, the simultaneous implantation of tumor cells into tens or hundreds of larvae enables parallel *in vivo* testing of multiple anti-cancer drug candidates on a scale that is unattainable using traditional mouse models. It is also noteworthy that most genes associated with human cancers have counterparts that are conserved in zebrafish, as are signaling pathways regulating cell proliferation, migration, and differentiation (Howe et al., 2013).

Another classic example of the application of zebrafish in oncology is the identification and characterization of anti-angiogenic drugs. The larval zebrafish model played a pivotal role in rediscovering the thalidomide’s anticancer mechanism by demonstrating its strong inhibitory effect on tumor angiogenesis through suppression of VEGF signaling. This effect was demonstrated in studies of blood vessel development in zebrafish (Yabu et al., 2005). These findings have helped establish thalidomide and its derivatives as therapeutic agents for neoplasms that depend on neovascularization, such as multiple myeloma. This confirms the value of the zebrafish model in identifying and validating clinically relevant anti-cancer drugs.

Importantly, the limitations of the zebrafish oncology model should be understood in the context of its intended use. This model is particularly well suited for rapid *in vivo* evaluation of tumor behavior, angiogenesis, metastatic potential, and comparative drug sensitivity, and it offers major advantages in throughput, visualization, cost, and experimental speed. Certain aspects of human oncology, including long-term tumor progression, fully mature immune responses, and some microenvironment-dependent processes, may require complementary confirmation in mammalian systems. Likewise, species-specific differences in temperature, pharmacokinetics, and tissue architecture should be taken into account during data interpretation. Nevertheless, these considerations primarily define the translational framework of the model rather than its weakness, and they strongly support the use of zebrafish as an efficient, informative, and ethically favorable platform for early-stage oncology research and treatment prioritization.

## Toxicology

The Polish Zebrafish Society recommends the use of zebrafish in toxicological research as a model facilitating early identification of substance toxicity. The zebrafish model is applied in the fields of food safety, and environmental and pharmaceutical toxicology, particularly in screening studies where large numbers of compounds require rapid safety assessment. A major advantage of this model is its close similarity to human physiology and metabolism, increasing the predictive value of toxicological outcomes (Batir-Marin et al., 2025; Hou et al., 2023; Zhao et al., 2024).

In the early stages of drug development, zebrafish can be used to assess the acute toxicity, as well as the teratogenic and embryotoxic effects, of tested compounds. Their rapid development also enables evaluation of embryotoxicity, including developmental delays and mortality. This allows potentially hazardous substances to be identified early, before progressing to more costly and ethically demanding tests involving larger laboratory animals or humans (Bauer et al., 2021; Dubińska-Magiera et al., 2016; Hamm et al., 2024; Miao et al., 2025; Shen and Zuo, 2020).

In toxicology, the zebrafish is employed as a full alternative to classical mammalian tests. While traditional rodent assays determine LD50 values (lethal dose), the Fish Embryo Acute Toxicity Test (FET, OECD TG 236) (Hamm et al., 2024; Kalueff et al., 2016; OECD, 2025) is based on the determination of LC50 values (lethal concentration) over a 96-h exposure period. These endpoints are not directly equivalent but provide comparable information on acute toxicity, enabling early hazard identification without the use of adult vertebrates. Importantly, LC50 values derived from zebrafish embryos are primarily used for screening and prioritization rather than direct regulatory substitution of mammalian LD50 data. At the same time, embryonic development in zebrafish enables evaluation of teratogenic and embryotoxic potential through direct observation of organogenesis and identification of morphological abnormalities induced by pharmaceuticals or chemical contaminants. This provides insight into the processes involved in the response, such as oxidative stress, apoptosis, and endocrine disruption (Jiang et al., 2015; Zhou et al., 2020). Zebrafish are also commonly used in the environmental risk assessment of new human drugs.

Zebrafish embryos offer several advantages that facilitate high-throughput screening, such as embryo transparency and a regular, small size. They can be automatically dispersed and maintained in a standard, multi-well plate format that is compatible with laboratory equipment for solvent and compound delivery, incubation, imaging and analysis. Techniques such as computerised readers and robotic microscopes allow for the automated detection of embryo orientation and the identification of specific areas of interest (e.g., the heart, brain or vessels, etc.). Combined with appropriate software and artificial intelligence, these features enable the rapid and detailed analysis of pharmacological, toxicological, and genetic effects *in vivo* (Bassi et al., 2025; Gierten et al., 2020; Letamendia et al., 2012; Lubin et al., 2021).

As a result of zebrafish’s potential, the scientific community and regulatory agencies recognize this model as an important vertebrate alternative to rodent and rabbit models. Implementing standardized protocols and approaches substantially reduces the number of mammals used in toxicity testing, decreases experimental costs and analysis time, and ensures compliance with current ethical and regulatory requirements. The FET assay has been incorporated into OECD guidelines as an accepted alternative method for chemical safety assessment. The Food and Drug Administration (FDA, USA) acknowledges the zebrafish as a tool for investigative and early-screening, preclinical procedures in the drug approval process. The European Medicines Agency (EMA) has also expressed strong interest in this model, as evidenced in preliminary regulatory reviews (Ball et al., 2025; Cassar, 2022; European Medicines Agency, 2018).

In line with the 3Rs principle, zebrafish should be recommended over rodent models whenever the scientific objective can be achieved using early whole-organism vertebrate assays that provide reliable and reproducible endpoints with lower ethical cost and higher throughput. This applies particularly to early hazard identification, acute toxicity assessment, developmental and embryotoxicity testing, teratogenicity screening, endocrine-disruption screening, and prioritization of large compound libraries before mammalian studies. In these contexts, zebrafish provide rapid and informative *in vivo* readouts while substantially reducing mammalian use.

Zebrafish can also contribute to chronic and reproductive toxicity research, especially in long-term exposure designs, fertility and reproductive performance studies, endocrine-related reproductive endpoints, and mechanistic investigations of developmental or transgenerational effects. However, they should not currently be regarded as a universal replacement for rodent assays when regulatory frameworks require mammalian data or when complex mammalian-specific physiology, pregnancy, lactation, multigenerational endpoints, or long-term systemic organ toxicity must be assessed. Species-specific differences in exposure conditions, pharmacokinetics, metabolism, and tissue complexity should also be considered when interpreting results. Therefore, the Polish Zebrafish Society recommends the use of zebrafish primarily within a tiered toxicological testing strategy, where they can replace or reduce selected rodent experiments at early and intermediate stages, filter compounds before mammalian testing, refine experimental design, and strengthen implementation of the 3Rs while preserving translational relevance.

## Neurology and neuropsychiatry

The Polish Zebrafish Society recommends using zebrafish as a model for analysing the mechanisms underlying epilepsy, anxiety disorders, depressive disorders, addiction and cognitive functions in neurological and neuropsychiatric research, including neuropsychopharmacology. Larval models are particularly valuable because they allow high-throughput behavioral and pharmacological analyses while maintaining high ethical standards. Experiments conducted on zebrafish larvae up to 5 dpf are recognized as an acceptable alternative to studies performed on adult vertebrates, enabling the investigation of psychoactive substances without significantly compromising animal welfare (Costalonga Rodrigues et al., 2025; Hillman et al., 2026).

Zebrafish exhibit a high degree of conservation of neurological and neuroendocrine pathways compared to humans (Kalueff et al., 2016; Stewart et al., 2012). This includes the presence of the hypothalamic–pituitary–interrenal (HPI) axis, which is homologous to the mammalian hypothalamic–pituitary–adrenal (HPA) axis, with conserved cortisol synthesis as the primary stress hormone (Alsop and Vijayan, 2009). Additionally, zebrafish possess well-organised neurotransmitter systems, including serotonergic, dopaminergic, glutamatergic and GABAergic pathways (Panula et al., 2006).

Epilepsy research is an important application of the zebrafish model. Seizures can be induced in larvae using the drug pentylenetetrazole, providing a reliable alternative to seizure assays used in mammals. In parallel, genetic epilepsy models have been developed based on mutations in genes homologous to human epileptogenic genes. One such gene is *scn1lab*, which corresponds to the human SCN1A gene that is associated with Dravet syndrome. Mutations in SCN1A in humans lead to impaired function of inhibitory interneurons and disruption of excitatory–inhibitory balance, and *scn1lab* mutants recapitulate these mechanisms by exhibiting reduced GABAergic signaling, spontaneous and stimulus-induced seizures, and abnormal neuronal activity patterns (Baraban et al., 2013; Dinday and Baraban, 2015).

These mutants exhibit spontaneous or inducible seizures, altered neuronal activity, and behavioral phenotypes corresponding to severe forms of epilepsy in humans (Baraban et al., 2007). Importantly, both PTZ-induced and *scn1lab*-based models enable assessment of neuronal network dysfunction using electrophysiological recordings and calcium imaging approaches, providing direct insight into seizure dynamics. While these models do not fully capture the complexity of human epilepsy, particularly chronic disease progression and higher-order brain functions, they faithfully reproduce core mechanisms of neuronal hyperexcitability and circuit dysfunction.

The serotonergic system, which encompasses enzymes that synthesize serotonin, transporters and receptors, performs functions in zebrafish that are analogous to those observed in humans. These functions include the regulation of mood, anxiety, impulsivity, sleep and social behaviors. The similarity of the neurochemistry and neurophysiology of the two species ensures that zebrafish behavioral models reflect key elements of human neuropsychiatric and neurodegenerative disorders, displaying high predictive value (Stewart et al., 2012).

Studies of anxiety-related behaviors in zebrafish employ standardized behavioral assays performed on larvae at approximately 5 dpf (Kalueff et al., 2016; Stewart et al., 2012). These assays include open-field tests (thigmotaxis), light–dark preference tests, and responses to sudden changes in illumination (light-dark challenge assay). Under these conditions, anxiolytic compounds reduce behaviors interpreted as anxiety-related, such as the avoidance of open spaces or darkness, in a manner comparable to effects observed in classical rodent anxiety models (Blaser and Gerlai, 2006; Maximino et al., 2010).

Zebrafish can also be used to analyze social behaviors and inter-individual interactions. The shoaling behavior and social preference of older larvae and juvenile fish can be assessed, enabling the evaluation of genetic and pharmacological influences on social (Dreosti et al., 2015; Hoffman et al., 2016) function. Such assays have been applied to model neurodevelopmental conditions relevant to autism spectrum disorders and to identify compounds that improve social phenotypes in genetically sensitized zebrafish lines. In addition, exposure to compounds affecting central nervous system signaling can alter these behaviors, further supporting the utility of zebrafish for translational social neuroscience research (Dreosti et al., 2015; Geng et al., 2023). The application of these models has enabled the identification and characterization of therapeutic compounds, the efficacy of which has been confirmed in subsequent mammalian and human studies. High-throughput phenotypic screening in zebrafish epilepsy models (including PTZ and *scn1lab*) is typically performed in multi-well formats using automated locomotor tracking and/or electrographic readouts (local field potentials) to quantify seizure activity (Dinday and Baraban, 2015). Compounds are first identified as “hits” based on a significant reduction of seizure-like behavior (e.g., high-velocity burst movements) and normalization of electrographic signatures compared to untreated mutants. One classic example is clemizole, which was identified in a high-throughput pharmacological screening process as a potent suppressor of seizures in the *scn1lab* zebrafish model of Dravet syndrome (Baraban et al., 2013). Following primary screening, candidate compounds undergo a structured validation pipeline in zebrafish, including (i) confirmation of efficacy across independent assays (behavioral and electrophysiological), (ii) dose–response analysis to establish therapeutic windows, (iii) assessment of toxicity and off-target behavioral effects, and (iv) testing in complementary seizure paradigms (e.g., PTZ-induced models). Mechanistic validation is further supported by pharmacological profiling (e.g., serotonergic or GABAergic modulation) and, where possible, genetic interaction studies. Importantly, promising candidates identified in zebrafish are subsequently validated in rodent models and, in some cases, advanced to clinical evaluation, demonstrating the translational relevance of this pipeline. Notably, zebrafish-based screening has contributed to the identification and evaluation of multiple classes of compounds with relevance to Dravet syndrome. These include serotonergic modulators such as fenfluramine (Sourbron et al., 2016), as well as non-traditional compound classes identified through phenotype-based screening approaches, such as synthetic cannabinoids (Griffin et al., 2020). Together, these findings highlight the ability of zebrafish assays not only to validate known therapeutic mechanisms but also to uncover unexpected anti-seizure candidates beyond classical anti-epileptic drug families, supporting their utility for precision therapy discovery.

In neuropsychiatric research, classical serotonergic drugs such as fluoxetine and citalopram induce behavioral effects in zebrafish that are consistent with their antidepressant and anxiolytic actions in humans. The translational relevance of these findings is supported by studies conducted both in adult fish and in larvae at 5 dpf (Airhart et al., 2007; Egan et al., 2009; MacRae and Peterson, 2015; Wong et al., 2013).

Zebrafish provide a particularly useful vertebrate system for studying conserved neurobiological processes that underlie seizure susceptibility, stress responsivity, and anxiety-related behavior. Their main strength lies in the possibility of combining genetic manipulation, live imaging, and quantitative behavioral readouts in a relatively rapid and scalable experimental setting. At the same time, interpretation should remain appropriately cautious, especially when extending findings to complex human neurological and psychiatric conditions. While zebrafish models capture important aspects of neuronal hyperexcitability, stress-axis regulation, and anxiety-like responses, they do not fully reproduce the full clinical and cognitive complexity of disorders such as chronic epilepsy, severe affective disease, or higher-order anxiety syndromes in humans. For this reason, zebrafish are particularly valuable for mechanistic studies, early functional phenotyping, and comparative pharmacological screening, whereas selected questions related to long-term disease course, complex emotional states, or advanced cognitive dysfunction may still require complementary validation in mammalian systems.

## Metabolic diseases

The Polish Zebrafish Society recommends the use of zebrafish in research on metabolic diseases, including obesity, dyslipidemia, and diabetes, non-alcoholic fatty liver disease, storage diseases, as well as in studies of metabolic disorders associated with cardiovascular disease. Zebrafish are a well-established model for investigating the pathogenesis and treatment of type 1 and type 2 diabetes, obesity, hepatic steatosis, and lipid metabolism disorders (Angom and Nakka, 2024; Benchoula et al., 2019; Vivekaa et al., 2025; Yang et al., 2025). Both larvae and adult zebrafish are recommended for mechanistic studies, early-stage disease modeling, and high-throughput *in vivo* screening, depending on the disease process under investigation. Larval zebrafish are particularly recommended for studies focused on early metabolic dysregulation, insulin signaling, glucose handling, and drug screening. Adult zebrafish, on the other hand, are more appropriate for investigations of long-term metabolic phenotypes, including obesity, dyslipidemia, hepatic steatosis, and vascular alterations induced by dietary interventions.

The *in vivo* visualization of organs important for metabolism, and a high degree of conservation of metabolic pathways between zebrafish and humans. Zebrafish retain homologous hormones and regulatory mechanisms controlling glucose and lipid homeostasis, including insulin, glucagon, nuclear receptors, and signaling pathways governing appetite and lipid storage. These features make the model highly suitable for representing core aspects of human metabolism and early responses to pharmacological and dietary interventions (Elo et al., 2007; Mullapudi et al., 2018; Toyoshima et al., 2008).

In type 2 diabetes research, insulin resistance models have been developed in zebrafish larvae by exposing them to elevated glucose concentrations, diabetogenic factors such as glucocorticoids (for example, dexamethasone), or exogenous insulin, often in combination with genetic manipulations that increase susceptibility to hyperglycemia and disturbances in carbohydrate metabolism (Nam et al., 2021; Salehpour et al., 2021; Titialii-Torres and Morris, 2022). While these models do not capture the full systemic complexity of human type 2 diabetes, they display features of insulin resistance and impaired glucose metabolism and can be used to evaluate the efficacy and toxicity of antidiabetic drug candidates *in vivo* (Nam et al., 2021; Salehpour et al., 2021). More than 70% of carbohydrate/glucose metabolic genes are conserved across zebrafish, frog, chicken, mouse, and human, and 57% of them are linked to human type 2 diabetes (Mullapudi et al., 2018; Zhang et al., 2018). Zebrafish share key carbohydrate metabolism genes and pathways, including insulin secretion, insulin resistance, and adipocytokine signaling, at levels similar to humans (Md Razip et al., 2022; Sanni et al., 2023; Zang et al., 2017). However, larval models do not fully recapitulate obesity-driven chronic adipose inflammation and long-term, age-dependent progression of metabolic dysfunction (Cao et al., 2023; Mullapudi et al., 2018; Van De Venter et al., 2020; Zhang et al., 2018).

The zebrafish has also been established as a robust model of type 1 diabetes mellitus based on the selective ablation or dysfunction of pancreatic beta cells. Transgenic lines expressing nitroreductase under the control of the insulin promoter enable inducible and highly specific destruction of beta cells upon exposure to metronidazole, resulting in rapid and reproducible insulin deficiency and hyperglycemia. These larval models recapitulate key pathophysiological features of type 1 diabetes, including loss of endogenous insulin production, impaired glucose homeostasis and activation of compensatory metabolic pathways. They also allow real-time, *in vivo* visualization of pancreatic islet structure and regeneration (Curado et al., 2007; Pisharath et al., 2007). Such systems have been widely used to study beta cell loss and regeneration, immune-independent mechanisms of diabetes, and to screen for small molecules that promote beta cell survival or regeneration under conditions that are directly relevant to type 1 diabetes.

Zebrafish are also used as a model for storage diseases caused by a deficiency of an enzyme responsible for breaking down substances. Examples include peroxisomal disorders, lysosomal, and glycogen storage diseases, and mitochondrial enzyme deficiencies. These disorders are often inborn metabolic disturbances that lead to pathological accumulation of substances such as lipids, glycogen, or glycoproteins, resulting in cell and tissue dysfunction and damage.

The zebrafish model provides in-depth insight into metabolic pathways, such as atypical vesicle trafficking, impaired autophagy, dysregulated signalling pathways, altered calcium homeostasis and mitochondrial dysfunction (Boustany, 2013; Parenti et al., 2021). For instance, the zebrafish model of Fabry disease, which is caused by a lack of alpha-galactosidase A activity due to a gene mutation, reveals further aspects of pathomorphology. Accumulation of globotriaosylceramide (Gb3) in lysosomes leads to organ failure. Results obtained using the zebrafish model show an independent mechanism of kidney damage due to mitochondrial alterations and disturbances in glycolysis, and galactose metabolism (Elsaid et al., 2023).

Due to their translational relevance, zebrafish serve as a gene knockout model, enabling the analysis of metabolic pathways, compensatory mechanisms and drug testing (Migocka-Patrzałek et al., 2020; Stefanik et al., 2024; T. Zhang and Peterson, 2020). The zebrafish has also been established as a valuable model for studying obesity and dyslipidemia. Adult fish subjected to long-term overfeeding or high-fat and high-cholesterol diets develop obesity and lipid disorder phenotypes comparable to those observed in mammals. Diets enriched in cholesterol induce lipid accumulation in the walls of blood vessels and lead to atherosclerosis-like changes. Meanwhile, key molecular pathways related to lipoprotein metabolism, inflammation and lipid oxidation remain conserved, functioning analogously to those in humans. Consequently, the zebrafish represents a useful tool for investigating the pathogenesis of cardiovascular diseases associated with metabolic disorders and for testing dietary and pharmacological interventions aimed at improving lipid profiles and vascular function (Fang et al., 2014; Karimzadeh et al., 2025; Stoletov et al., 2009; Zang et al., 2018).

In summary, zebrafish models in the metabolic diseases are particularly informative for studying beta cell loss and regeneration in diabetes, lipid handling and vascular lipid deposition in dyslipidemia, intracellular substrate accumulation in storage diseases, and early metabolic–cardiovascular interactions that precede overt organ damage. Some aspects of metabolic disease should, however, be interpreted with caution in zebrafish. In particular, the long-term progression of chronic metabolic disorders, the full complexity of endocrine regulation, and selected complications involving adult organ physiology may not always be fully reproduced, especially in larval models. Differences in nutritional physiology, lifespan, body composition, and systemic metabolic integration should also be taken into account when extrapolating findings to humans. These considerations are especially relevant for disorders in which prolonged disease duration and multisystem interactions play a major role. Nevertheless, such limitations mainly define the boundaries of interpretation and do not reduce the substantial value of zebrafish for mechanistic and translational metabolic research.

Accordingly, the Polish Zebrafish Society recommends the use of zebrafish when research questions require *in vivo* visualization, genetic tractability, rapid phenotyping, or ethically refined screening approaches, while acknowledging that advanced endocrine complexity and long-term disease consequences may require validation in mammalian systems. From the perspective of the 3R principles, zebrafish models can contribute substantially to replacement and reduction strategies as a complementary model or, where appropriate, a partial replacement at early and intermediate stages of research, particularly for hypothesis generation, pathway analysis, and candidate drug prioritization prior to validation in rodent models.

## Immunology

The Polish Zebrafish Society recommends the use of zebrafish in studies of immune responses, including the investigations of inflammatory mechanisms and the development of high-resolution models to study host-pathogen interactions on molecular, cellular and populational levels during bacterial and viral infections. A major feature of this model lies in the ontogeny of the immune system. During the first week of life, zebrafish larvae possess only a functional innate immune system, with components of adaptive immunity developing several weeks later (Lam et al., 2004). This temporal separation offers a dual perspective. It is a significant experimental advantage for the selective investigation of innate immune mechanisms such as neutrophil and macrophage responses without interference from adaptive immunity. However, it also represents a limitation for researchers aiming to study complex lymphocyte-mediated responses or long-term immunological memory in larval stages of zebrafish.

The transparency of zebrafish larvae, coupled with an extensive toolkit of transgenic lines with fluorescently labelled immune cells, enables the direct visualization of leukocyte migration, inflammatory dynamics, and host–pathogen interactions in living organisms. Widely used lines include *Tg(mpx:GFP)* for monitoring neutrophils (Renshaw et al., 2006), and *Tg(mpeg1:mCherry)* for macrophages (Ellett et al., 2011). These models are commonly employed in neuroinflammation, intestinal inflammation, skin inflammation and steatohepatitis studies, enabling the monitoring of immune cell infiltration *in vivo* and the evaluation of the effects of immunomodulatory and anti-inflammatory agents in real time.

An important application of the zebrafish immunological model is research on pathogenesis and therapeutic interventions for bacterial and viral infections. A classic example is the infection of zebrafish larvae with *Mycobacterium marinum*, which is closely related to *Mycobacterium tuberculosis*. In infected larvae, granuloma-like structures are formed, and both the infection progression and the host responses can be analyzed in real time thanks to the organism’s transparency. This model has enabled the identification of key pathogen virulence factors and host defense mechanisms relevant to tuberculosis pathogenesis. It has also been applied in high-throughput screening efforts aimed at identifying novel antitubercular drugs. The zebrafish model of *M. marinum* infection effectively recapitulates the hallmark pathological features of human tuberculosis, specifically the formation of organized granulomas consisting of aggregated macrophages that undergo epithelioid transformation (Cronan et al., 2016). However, the model has specific limitations; the larval stage lacks the T-cell-mediated responses that characterize mature human granulomas, and the lower physiological temperature of zebrafish (28 °C–30 °C compared to 37 °C in humans) may influence the metabolic rate of the pathogen and the kinetics of the immune response (Tobin and Ramakrishnan, 2008). Despite these differences, the model’s strength lies in its ability to provide high-resolution, real-time insights into the earliest stages of infection and granuloma initiation, which are largely inaccessible in mammalian models (Ramakrishnan, 2012).

Apart from studying tuberculosis, similar approaches are used to study infections caused by other pathogens including *Salmonella* Typhimurium and *Staphylococcus aureus*. This highlights the broad applicability of the zebrafish model in the fields of immunology and microbiology (Meijer and Spaink, 2011).

Zebrafish have recently become a popular model for studying cell-autonomous immunity, particularly the way in which individual cells utilize the autophagic response to eliminate intracellular pathogens. By using the *Tg(CMV:GFP-Lc3)* reporter line, researchers can track the recruitment of the autophagy marker LC3 to bacterial vacuoles in real time (He et al., 2009). Apart from *M. marinum*, studies involving *S. aureus* and *S.* Typhimurium have utilized this model to demonstrate how various canonical and non-canonical autophagy pathways influence host-pathogen interactions (Masud et al., 2019; Prajsnar et al., 2021; Zhang et al., 2019). These mechanisms are typically analyzed through high-resolution microscopy combined with the knockdown of pathway-specific genes, which provides insight into how the host handles infections at the subcellular level (Michno et al., 2025). Ultimately, while zebrafish cannot fully replace mammalian models for all facets of human immunology including those requiring complex mammalian tissue architecture or 37 °C-specific kinetics, they serve as a critical bridge between *in vitro* assays and mammalian *in vivo* studies, fulfilling the 3Rs by refining experimental design and reducing the reliance on higher vertebrates.

## Cardiology

The Polish Zebrafish Society recommends the use of zebrafish in cardiovascular research, including both basic studies of heart development and function and safety pharmacology assessments, particularly those addressing the cardiotoxicity and proarrhythmic potential of drugs (Langheinrich et al., 2003; Maciag et al., 2022; Milan et al., 2003). Despite having a simpler anatomical structure, the zebrafish heart exhibits important physiological similarities to the human heart, particularly regarding electrophysiology and the mechanisms of contractility (Stainier, 2001).

The presence of homologs of key cardiac ion channels, including the hERG channel, results in zebrafish respond to QT-prolonging drugs in a way that is comparable to humans. This has been demonstrated in pharmacological studies conducted in embryos, larvae, and adult fish (Langheinrich et al., 2003; Milan et al., 2003).

In preclinical studies, zebrafish larvae are used to assess the effects of compounds on heart rhythm, heart rate, and conduction disturbances using high-speed video imaging and quantitative analytical approaches. Evaluating these parameters enables the early identification of compounds with arrhythmogenic or cardiotoxic potential, avoiding the need to progress to mammalian studies or clinical trials. Meanwhile, the transparency of larval tissue enables the simultaneous evaluation of cardiac hemodynamics and structural changes to the heart and vasculature (Langheinrich et al., 2003; Maciag et al., 2022).

Importantly, zebrafish cardiac assays have already provided several concrete and translationally relevant examples of successful applications. In an early landmark study, compounds known to induce repolarization abnormalities and QT prolongation in humans were shown to consistently trigger bradycardia and atrioventricular block in zebrafish, and 22 of 23 clinically relevant compounds were identified as positive in the assay, supporting the utility of zebrafish for early detection of proarrhythmic liability (Milan et al., 2003). Beyond toxicological screening, zebrafish-based cardiac assays have also contributed to mechanistic discovery. Using a drug-sensitized screening approach, 15 genes affecting cardiac repolarization were identified, including GINS3, whose human ortholog mapped to a locus associated with QT interval in human genome-wide association studies, demonstrating the value of this model for uncovering conserved regulators of myocardial electrophysiology (Milan et al., 2009). Finally, zebrafish have also proven useful in therapeutic discovery. In a zebrafish model of doxorubicin-induced cardiomyopathy, screening of 3,000 compounds identified visnagin and diphenylurea as cardioprotective molecules that rescued cardiac performance and circulatory defects, with subsequent validation in mice, highlighting the translational potential of zebrafish assays for the identification of candidate cardioprotective agents (Liu et al., 2014). Together, these studies show that zebrafish cardiac assays can support not only the detection of cardiotoxic and arrhythmogenic compounds, but also mechanistic discovery and the identification of therapeutically relevant small molecules.

The zebrafish has also been extensively used to study the mechanisms underlying cardiac disease and regeneration. Adult zebrafish possess the ability to regenerate cardiac muscle following injury or myocardial infarction by dedifferentiating and proliferating existing cardiomyocytes. This regenerative capacity, which is absent in adult mammals, makes zebrafish a unique model for investigating heart regeneration and identifying molecular pathways that may represent potential therapeutic targets for human cardiovascular disease (Jopling et al., 2010).

Certain aspects of cardiovascular research should be interpreted with appropriate caution in zebrafish. While the model captures many conserved features of cardiac electrophysiology, myocardial contraction, and drug-induced functional disturbances, it does not fully reproduce the structural and physiological complexity of the human cardiovascular system, particularly in relation to the four-chambered heart, coronary circulation, and chronic remodeling processes. These considerations are especially relevant for disorders requiring long-term observation of adult cardiac pathology or complex systemic hemodynamic interactions. Accordingly, findings from zebrafish are best interpreted as highly informative within the context of early functional analysis and pathway discovery, and as a strong basis for further investigation when broader cardiovascular complexity must be addressed.

## Genetic models of human diseases

The Polish Zebrafish Society recommends use of zebrafish as a genetic model for human diseases, particularly monogenic disorders and conditions resulting from the dysregulation of key molecular pathways. Zebrafish models are particularly well suited to address research questions such as: whether a candidate human disease variant is pathogenic *in vivo*, how perturbations in conserved molecular pathways affect organ development and function, and which genetic or pharmacological modifiers can rescue disease phenotypes. Approximately 70% of human protein-coding genes have at least one zebrafish ortholog, and this proportion increases to over 80% for genes associated with human disease, with high conservation of core developmental and signaling pathways. In contrast to highly inbred laboratory mouse strains, zebrafish lines typically maintain greater genetic diversity, which can more closely reflect the heterogeneity observed in human populations (Howe et al., 2013; Kettleborough et al., 2013; Navratilova et al., 2009). Due to the high degree of genetic homology between zebrafish and humans, targeted mutations in orthologous genes enable reproduction of disease phenotypes observed in patients (Lieschke and Currie, 2007; MacRae and Peterson, 2015). The use of zebrafish models aligns with concerns regarding genetic variability as a confounding factor in preclinical-to-clinical translation. By enabling studies across diverse genetic backgrounds and rapid generation of multiple mutant alleles, zebrafish provide a platform to assess the robustness and reproducibility of disease phenotypes and therapeutic effects prior to costly mammalian or clinical studies (Balik-Meisner et al., 2018; Brown et al., 2012).

The zebrafish is a valuable experimental vertebrate model for studying rare disorders and unknown gene variants with potentially pathological consequences. The availability of genetic tools such as next-generation sequencing (NGS), combined with the relatively easy generation of mutants using CRISPR-Cas9 technology, facilitates high-throughput functional screening of candidate genes and variants. Zebrafish are also useful models for understanding human variant pathology due to the ease with which observable phenotypes linked to gene disruption can be identified during its development. Functional assays performed on zebrafish can determine the potential pathogenicity of variants of uncertain significance. Furthermore, this animal model allows for molecular and biochemical analysis and preclinical drug testing. The zebrafish model supports the experimental validation of human genetics, permitting the identification of novel genetic variants associated with rare diseases (Angom and Nakka, 2024; Choi et al., 2021; Lin et al., 2025; Sarosiak et al., 2025; Varshney et al., 2016). The zebrafish model is particularly useful in the early stages of research, allowing the rapid determination of gene function, the assessment of mutation consequences, and the *in vivo* screening of potential pharmacological and gene-based therapeutic strategies (Lieschke and Currie, 2007). In research practice, targeted mutagenesis techniques, including CRISPR/Cas9, are widely employed to generate zebrafish lines carrying mutations corresponding to human pathogenic variants (Hwang et al., 2013). This enables the modelling of a wide range of genetic diseases.

The zebrafish has been applied in modelling muscular and locomotor diseases. These include myopathies (e.g., Duchenne and limb-girdle), sarcopenia (e.g., age-related muscle weakness and diet-induced disturbances), motor neuron diseases (e.g., spinal muscular atrophy, SMA), and movement disorders (e.g., Charcot–Marie–Tooth and Parkinson’s disease) (Garg and Geurten, 2024; Plantié et al., 2015; Vaz et al., 2018; Zhao et al., 2024). The Duchenne zebrafish model is generated through targeted mutations in the dystrophin gene, yielding phenotypic manifestations characteristic of Duchenne muscular dystrophy. These manifestations include pronounced muscle weakness, deficits in locomotor performance, and progressive degeneration of skeletal muscle fibers (Bassett et al., 2003). Several protocols and approaches have been developed specifically to test motor behavior, muscle fiber structure and performance. The touch-evoked escape response and locomotor assays are used to quantify acceleration and swimming activity, providing quantitative data regarding muscle performance and function during early development (Sztal et al., 2016). The high-resolution 3D microscopy is usually used to analyze muscle fiber structure (Van Meer et al., 2025). The force transducer-based contractions techniques, where larvae are electrically stimulated, are used to measure contractile properties, and kinetics of muscles (Shorten et al., 2007; Sloboda et al., 2013). Living biosensor models that can express fluorescent reporter proteins under the control of muscle-specific promoters or responsive elements are available. Such an approach allows for dynamic processes such as muscle cell differentiation, contraction and motor protein turnover to be observed (Moro et al., 2013; Tesoriero et al., 2023). Fluorescent muscle reporters can be used for rapid whole-embryo screening and to monitor global muscle activity over time. Analysis can be performed using widefield and/or epifluorescent time-lapse microscopy. Confocal laser scanning microscopy enables more detailed analysis at the level of individual cells and fibers, as well as 3D reconstructions (Facchinello et al., 2016; Moro et al., 2013; Tesoriero et al., 2023). Spinning disk confocal microscopy enables the imaging of dynamic processes such as fiber growth or Ca^2+^ signal dynamics thanks to its reduced phototoxicity (Webb et al., 2012). Two-photon microscopy is used for deep tissue imaging of internal muscles and neuromuscular junctions in later stages of zebrafish development when they become less transparent (Oralová et al., 2019). Fluorescence resonance energy transfer (FRET)-based molecular biosensors are available for studying protein activity in live zebrafish embryos (Kardash et al., 2011). Muscle differentiation and the quantification of pathway activity during myogenesis can be achieved using transgenic biosensor animals. This approach involves expressing fluorescent proteins under the control of signaling pathway-responsive cis-elements (Facchinello et al., 2016; Moro et al., 2013). These models have been used to test therapeutic strategies, including pharmacological interventions and gene-therapy approaches, aimed at restoring protein function or compensating for its absence.

Genetic zebrafish models are also widely applied to the study of metabolic and developmental disorders where mutations in a single gene can disrupt the function of organs such as the liver, pancreas or kidneys. Larval transparency allows direct assessment of developmental and functional abnormalities in these organs, while their high fecundity and rapid development enable the analysis of large cohorts within short timeframes. Consequently, the use of zebrafish as a genetic disease model contributes to accelerated identification of disease-causing genes and supports the development of precision medicine approaches that integrate genetic data with functional *in vivo* phenotyping (Lieschke and Currie, 2007; MacRae and Peterson, 2015).

In genetic disease research, the main point requiring careful interpretation is not the utility of the model itself, but the extent to which a given zebrafish phenotype reflects the full human clinical presentation. Although disruption of orthologous genes often reveals conserved developmental and molecular mechanisms, the resulting phenotype may represent only selected components of the disease spectrum observed in patients. This is particularly relevant for disorders influenced by tissue-specific context, postnatal physiology, genetic background, or long-term progression. In addition, gene duplication in zebrafish and the presence of paralogous genes may complicate direct comparison with human monogenic conditions and should be considered during experimental design and data interpretation. For this reason, zebrafish findings are especially informative at the level of gene function, variant assessment, and early disease mechanism, while broader genotype-phenotype correlations may require integration with complementary mammalian or clinical data. Zebrafish should be considered the preferred first-line vertebrate model for gene function analyses, including loss-of-function and gain-of-function approaches, prior to the employment of mammalian systems. This strategy is fully aligned with the principles of the 3Rs as early-stage validation and screening in zebrafish can substantially reduce the number of mammalian experiments required and refine hypotheses before transition to more complex and ethically demanding models.

## Discussion

The efficiency of translation of preclinical drug candidates to approved therapeutics remains one of the most persistent failures in biomedical science. The overall preclinical-to-market failure rate exceeds 92%, a figure unchanged for three decades. The Quality of generated data was controversial leading to development of improved methodologies and strategies. Exploratory and preclinical studies cannot be conducted in humans, leaving only several experimental options. Among the available experimental approaches, *in vitro* systems have demonstrated the most significant limitations in this context. Classical 2D cell cultures have been widely used but for many years now it is accepted that the informational value of these studies is too limited to consider. A certain improvement was the development of 3D cultures, nonetheless the physiological dependencies or cell-cell effects are not tractable in this system.

A new development of NAMs (New Alternative Methodologies although other expansions of this acronym are also used) promises to address the basic issues associated with classical *in vitro* work. NAMs include organoids, organs on a chip and the likes. They do address some issues such as organ/organ interactions but do not reflect full organismal physiology, essential when studying the pharmacokinetics and metabolomics of drugs. The only viable alternative today is use of animal-based model organisms. These, however, do have a number of limitations compounding the experimental challenges. The most popular model organism by far is the laboratory mouse. Mouse models produce positive preclinical results in 90%–95% of studies, yet fewer than 8%–10% of those results translate to approved drugs. In oncology the figure falls to 3.5%. In inflammation and neurodegeneration the track record is substantially worse. This is not primarily a question of mouse biology being wrong, it is a question of study design, publication bias, sex bias (most mouse studies use male animals), lack of genetic diversity, and failure to reproduce the full complexity of human immunology and metabolism. Additionally, mouse-based studies are slow, costly and have a very significant administrative burden. The current legislation and ethical regulations, spurred by the very low “mouse-to-clinic” ratio, render this model increasingly impractical for many research applications (Begley and Ellis, 2012; Hutchinson and Kirk, 2011; Kola and Landis, 2004).

Therefore, use of zebrafish in preclinical studies has been systematically increasing, surpassing the use of mouse models in several research domains. It is very important to stress that zebrafish under 120 hpf, which do not swim or feed independently, are considered NAMs according to EU Directive 2010/63. Additionally, Federal Drug Administration (FDA) developed a draft on use NAMs in preclinical research, paving the way to use zebrafish in these types of studies (https://www.fda.gov/news-events/press-announcements/fda-achieves-year-1-goals-reducing-animal-testing-drug-development). Two key types of preclinical studies stand out. One of them is a high-throughput safety pre-filter and phenotypic hit identifier. In this domain zebrafish has proven to be today the model with the highest predictive value with studies focusing on drug cardiotoxicity benefiting most. The second preclinical, and even clinical beneficiary, are studies involving zebrafish cancer avatars. These strategies, currently irreplaceable by other approaches, are applied in clinics with direct benefits for the patients.

Therefore, we recommend use of zebrafish as the most productive preclinical pipeline for an early-stage filter reducing the number of compounds entering expensive and lower-throughput mammalian studies or cheap but uninformative 2D *in vitro* models.

### Practical framework for the implementation of zebrafish-based approaches in biomedical research

To support the broader and more systematic adoption of zebrafish in biomedical research, implementation should be approached through a structured, fit-for-purpose framework rather than by treating the model as a universal replacement for mammalian systems. The first step is to define the primary objective of the study, as zebrafish can serve different roles depending on the context, including early phenotypic screening, toxicity assessment, mechanistic studies, disease modeling, or preliminary therapeutic validation. Clear identification of the intended purpose is essential for selecting an appropriate experimental design and for determining how zebrafish-derived data should be integrated into the overall research workflow.

The second step is to align the biological question with the most suitable developmental stage and assay format. Zebrafish embryos and larvae are particularly advantageous for high-throughput applications, live imaging, reporter-based analyses, and early safety or efficacy screening. Their small size, optical transparency, and compatibility with multi-well platforms make them especially useful for studies requiring rapid and scalable phenotyping. In contrast, more complex physiological, behavioral, or long-term disease-related endpoints may require the use of older larvae or adult fish. Thus, implementation should be based not only on the disease area of interest, but also on the level of biological complexity needed to answer the specific question.

A further key requirement is the selection of robust and biologically relevant endpoints. Depending on the field of application, these may include survival, morphology, organ-specific toxicity, heart rhythm and rate, locomotor activity, inflammatory cell recruitment, reporter fluorescence, metabolic alterations, or tumor-related phenotypes. Importantly, endpoints should be selected on the basis of their translational relevance and reproducibility, rather than solely on technical convenience. Whenever possible, zebrafish assays should be designed to capture measurable phenotypes that are functionally linked to known disease mechanisms or therapeutic targets.

For practical implementation, assay standardization is critical. Each zebrafish-based protocol should include clearly defined experimental conditions, including developmental stage, exposure window, compound delivery method, exposure concentration, environmental parameters, exclusion criteria, and endpoint timing. Positive and negative controls should be incorporated routinely, and assay performance should be verified using reference compounds or interventions relevant to the intended application. Such standardization is necessary to ensure inter-study comparability, facilitate reproducibility, and improve confidence in the interpretation of zebrafish-derived findings across laboratories and institutions.

Equally important is the integration of zebrafish into tiered decision-making pipelines. In many settings, the zebrafish model is likely to be most valuable as an intermediate platform positioned between simple *in vitro* systems and more resource-intensive mammalian studies. In this role, zebrafish can be used to prioritize compounds, identify early toxicity signals, reveal biologically active phenotypes, and refine hypotheses before advancing to mammalian validation. However, the criteria for such progression should be predefined. A practical implementation strategy should therefore specify what constitutes a positive finding, which endpoints justify follow-up, and in which situations subsequent validation in mammalian models remains necessary. This is particularly important in research areas where systemic pharmacokinetics, adaptive immunity, organ-specific physiology, or late-stage pathology cannot be adequately modeled in early zebrafish life stages.

From an institutional perspective, successful implementation also requires investment in infrastructure, training, and reporting standards. Laboratories seeking to adopt zebrafish-based approaches should establish standardized husbandry and experimental procedures, ensure personnel training in zebrafish biology and phenotyping, and implement quality-control measures appropriate for the planned assays. In parallel, funding bodies, ethics committees, and regulatory stakeholders should recognize zebrafish as a scientifically valuable component of tiered preclinical workflows, particularly in contexts where the model can reduce mammalian use while preserving biological relevance. In this sense, implementation should not be viewed solely as a technical decision, but also as part of a broader strategy to improve experimental efficiency, translational prioritization, and alignment with the 3R principles.

The Polish Zebrafish Society can support the implementation of zebrafish-based approaches by providing scientific consultation on model selection, experimental design, endpoint choice, and interpretation of zebrafish-derived data. The Society can also facilitate contact with experienced laboratories, support standardization of protocols, promote training activities and workshops, and provide recommendations useful for grant applications, ethics-related documentation, and institutional implementation of zebrafish facilities. In this role, the Society may serve as a national advisory platform connecting researchers, institutions, ethics committees, and regulatory stakeholders interested in the responsible use of zebrafish in biomedical research.

Overall, effective integration of zebrafish into biomedical research requires a question-driven and application-oriented approach. When matched appropriately to the research objective, standardized experimentally, and embedded within a tiered validation strategy, zebrafish-based assays can provide a practical and scalable platform that supports both scientific discovery and more ethical preclinical research. A practical overview of major zebrafish applications, recommended uses, key readouts, advantages, and situations requiring mammalian validation is provided in Table 1.

**TABLE 1:** Practical integration of zebrafish into biomedical research workflows.

Research aim	Recommended zebrafish use	Key readouts	Main advantages	When mammalian validation is needed
Early drug screening	Primary *in vivo* screening	Survival, morphology, behavior, reporter activity	High throughput, whole-organism context, low compound demand	PK/PD, target engagement, dose translation
General toxicity	Early safety assessment	Mortality, malformations, edema, organ toxicity	Rapid vertebrate-level toxicity detection	Chronic, reproductive, regulatory toxicology
Cardiotoxicity	Functional safety screening	Heart rate, rhythm, AV block, circulation defects, edema	Optical accessibility, rapid cardiac phenotyping	Detailed electrophysiology, definitive translational confirmation
Neurobehavioral studies	Early screening and prioritization	Locomotion, seizure-like activity, anxiety-like behavior, sleep-related phenotypes	Scalable behavioral phenotyping	Complex cognition, mammalian-specific CNS responses
Inflammation	Mechanistic and screening platform	Immune-cell recruitment, inflammatory reporters, injury responses	Live imaging, fluorescent reporter lines	Adaptive immunity (unless adult zebrafish used), chronic immune pathology
Infectious disease	Host-pathogen and anti-infective studies	Pathogen burden, survival, immune responses, reporter activity	Real-time *in vivo* imaging, scalable assays	Full mammalian pathology, host specificity, adaptive immunity (unless adult zebrafish used)
Metabolic disease	Mechanistic and early intervention studies	Lipid accumulation, steatosis, glucose-related phenotypes, oxidative stress	Rapid imaging-based phenotyping	Long-term metabolic regulation, higher physiological complexity
Oncology/xenografts	Early tumor biology and drug-response studies	Proliferation, migration, invasion, angiogenesis	Fast *in vivo* tumor assays, low sample input	Long-term tumor progression, complex microenvironment
Target validation	Functional *in vivo* testing	Genetic or reporter-based phenotypes	Genetic tractability, efficient hypothesis testing	Species-specific target biology, tissue-level confirmation
Preclinical pipeline integration	Intermediate decision platform	Fit-for-purpose endpoints	Better prioritization, reduced mammalian use, 3R alignment	Regulatory and late-stage translational validation

The table summarizes major applications of zebrafish in biomedical research, the most relevant readouts, the main practical advantages of the model, and situations in which mammalian validation remains necessary (Howe et al., 2013).

## Summary

The zebrafish has firmly established itself as one of the most important alternative tools in biomedical research, currently representing the second most frequently used animal model in scientific research within the European Union after mice. Using embryos and larvae up to 5 days post-fertilization, in accordance with applicable legal regulations and the 3Rs principles, enables the genuine replacement of certain studies that were previously conducted in mammalian models. This leads to a substantial reduction in the overall number of higher vertebrates used in experimental research.

At the same time, this model significantly accelerates the development of new therapies by enabling the rapid, relatively cost-effective *in vivo* screening of large numbers of drug candidates in the early stages of preclinical research. The Polish Zebrafish Society therefore recommends that research institutions, pharmaceutical companies and regulatory agencies systematically incorporate the zebrafish-based approaches described in this document into their research standards.

In practice, this involves incorporating zebrafish embryo and larval assays into the evaluation of the efficacy and safety of newly developed biologically active substances, as well as promoting this model in research projects and grant applications. This approach provides valuable translational data due to the high genetic and physiological similarity between zebrafish and humans. It also aligns with global trends towards ethical, responsible and sustainable biomedical research. Therefore, integrating these recommendations contributes to more humane, efficient and innovative scientific research, ultimately benefiting medical progress and public health.
